# Truth

**Published:** 1890-04-12

**Authors:** 


### Words of Consolation.

### TRUTH.

What is Truth? This question was addressed to Christ by Pilate, and is one which every thinking person has asked himself from the earliest times to the present day. Before the coming of Christ upon earth, men could find no satisfying answer to the enquiry, but when he appeared, He announced that He was come to bear witness to the Truth and that He Himself was the Truth and the life everlasting. No man, said He, can go to the Father but by Me. Christ made the Truth known by taking on Himself the form of man and bearing witness by His words and deeds. Man being made in God's image should also bear witness to the Truth according to their position in life. Christ showed the truth of His ministry by His sufferings even unto death. The truth of inferior messengers have been tested by their sufferings also. They bring messages which are opposed to the natural heart of man so they are persecuted for righteousness' sake. Which of the prophets did not the fathers persecute, says St. Stephen at his trial; and they slew them which showed before of the coming of the Just One, and ended by becoming His betrayers and murderers. St. James also points out that we can take the Prophets who have spoken in the Name of the Lord for examples of suffering, patience, and affliction. Our Lord is the Great Example above all. The Truth to which He bore witness is the reward of all who bear witness to Him in their lives. "The Lord Himself is the portion of mine inheritance and of my cup," cries the Psalmist. No inferior reward could be a recompense. Then let us follow after the Truth in all things to gain this rich reward, this dwelling in Christ and Christ in us. We will ask ourselves first whether we know what is Truth. Have we taken Jesus for our Saviour? Do we earnestly believe in Him as the only Redeemer of our souls? Is our faith founded on this Rock and do we in trouble and suffering find comfort in this Sure Foundation?

This faith in the Truth must furthermore be shown in our lives, when by God's mercy He gives us back health and strength. We must avoid all that leads away from truth; we must hate a lie and Satan the father of lies. Our Lord tells us Satan was a liar from the beginning. We know how he deceived Eve and caused the punishment of the whole human race. He whispered a doubting question in her ear, "Yea, *hath* God said thou shalt not eat of the tree of knowledge?" She began to think if it really were so, and then to long for the forbidden fruit. Do we ever have doubts whispered in our ears, such as of the truth of Holy Scripture, or of the goodness of God, or of His mercy and kindness to His creatures? Turn away from such thoughts; do not harbour them for an instant. Pray earnestly to Him who is the Truth to guide you into all truth.

We all know the satisfaction and comfort we feel in dealing with one on whose word we can fully rely. This comfort and satisfaction are reflected from that God whose words can never fail, whose promises are sure and will endure for ever and ever.

